# Efficacy of prokinetic agents in diabetic gastroparesis comparing symptomatology and scintigraphy – An open-label trial

**DOI:** 10.22088/cjim.14.4.618

**Published:** 2023

**Authors:** Ravi Kant, Madhuri Pratti, Meenakshi Khapre, Poonam Yadav, Vandana Dhingra

**Affiliations:** 1 Department of Medicine All India Institute of Medical Sciences, Rishikesh, Uttarakhand, India; 2 Department of Community Medicine, All India Institute of Medical Sciences, Rishikesh, Uttarakhand, India; 3 College of Nursing, All India Institute of Medical Sciences, Rishikesh, Uttarakhand, India; 4 Department of Nuclear Medicine, All India Institute of Medical Sciences, Rishikesh, Uttarakhand, India

**Keywords:** Cinitapride, Diabetes mellitus, Gastroparesis, Gastric scintigraphy, Gastric emptying, Levosulpiride.

## Abstract

**Background:**

It is pertinent to objectively assess the severity of diabetic gastroparesis and tailor treatment accordingly. The current study was planned to document gastroparesis by gastric emptying scintigraphy (GES) objectively and see the effect of medications and diet control on clinical and GES after four weeks.

**Methods:**

A prospective, open-label randomized trial was conducted in the Department of Internal Medicine at a tertiary care teaching hospital over twelve months. Type 2 diabetic patients aged 18-65 years diagnosed with a case of delayed gastric emptying by gastric scintigraphy were included. All baseline GSCI was recorded, and then they were allotted to 3 groups – Group-1 (Levosulpiride 25mg once daily), group-2 (Cinitapride 1mg thrice daily), and Group-3 (Waitlisted control) by block randomization and followed-up weekly till four weeks. After four weeks duration, if not improved clinically, then Group-3 on diet and diabetic control only, were randomized into Levosulpiride 25mg once daily (Group 1), and Cinitapride 1mg Thrice daily (Group 2) for the next four weeks.

**Results:**

Forty confirmed cases with diabetic gastroparesis documented by Gastroparesis Symptom Cardinal Index (GCSI) scoring and later by Scintigraphy (GES) were included in this study. However, there was no statistically significant difference between the Levosulpiride and Cinitapride groups when all symptoms were taken into account. Levosulpiride was significantly more effective than Cinitapride in improving individual symptoms like nausea, vomiting, stomach fullness, and early satiety.

**Conclusion:**

Levosulpiride is better than Cinitapride in improving the symptoms of diabetic gastroparesis but no significant effect on gastric scintigraphy.

Diabetes Mellitus (DM) refers to a group of common metabolic disorders that share a hyperglycemia phenotype (1). The prevalence of DM has shown an upslope trend in the last two decades, both in developed and developing countries, with 30 million cases in 1985 to 415 million in 2017. IDF has projected an increasing DM trend to show an estimated prevalence of around 642 million people with diabetes by 2040 (2, 3). The chronic hyperglycaemic state, often accompanied by abnormally elevated advanced glycation end products (ages) in diabetic patients. It disturbs normal homeostasis of the human body resulting in various long-term (microvascular and macro-vascular) and short-term complications like diabetic ketoacidosis (DKA) and hyperosmolar hyperglycaemic state (HHS). Microvascular complications are usually diabetes-specific; macrovascular complications have underlying pathophysiologic features shared by diabetic and non-diabetic populations (2, 4). 

Diabetic neuropathy, a common microvascular complication of DM, occurs in about ~50% of individuals with long-standing DM. DM-related autonomic neuropathy can involve multiple organs such as cardiovascular, gastrointestinal, genitourinary, sudomotor system, etc (2, 4). Diabetic gastrointestinal autonomic neuropathy can affect any organ or segment of the digestive system, starting right from the esophagus extending to the large intestine through the stomach, gallbladder, pancreas small intestine. Gastroparesis is one of the clinically apparent important manifestations of gastrointestinal autonomic neuropathy characterized by delayed or abnormal gastric emptying without definite mechanical obstruction (5). 

Gastroparesis has protean etiologies, diseases like Hypo/hyperthyroidism, Post-viral, Autoimmune diseases, Secondary to medications such as opioids, TCA's, beta-blockers, CCB, Post-surgical Diabetes Mellitus, with its increasing prevalence, tops the list of common and important causes of Gastroparesis. Usual presenting complaints of DGP include – nausea, vomiting, early satiety, postprandial fullness, upper abdominal pain (6). 

DGP often gets complicated in the advanced stage with nutritional deficiency, loss of weight, serum electrolytes abnormality, bezoar formation, aspiration pneumonia, etc. Studies from tertiary academic medical centers have reported a higher prevalence of DGP among type 1 diabetics (40 %) than type 2 diabetics (10 – 20 %), whereas community prevalence was reported to be around 5 % for type 1 diabetics and 1 % among type 2 diabetics (6). Gastroparesis diagnosis is usually made based on the combination of symptoms suggestive of gastroparesis and objective evidence of delayed gastric emptying after ruling out gastric outlet obstruction. Objective assessment of delayed gastric emptying is the mandatory criteria for making a definite diagnosis of gastroparesis (4, 7). 

Gastric emptying scintigraphy is the most widely available and gold standard procedure for measuring delayed gastric emptying (8, 9). Initial management of gastroparesis starts with the restoration of fluids/electrolytes, nutritional supplementation, and diabetics optimization of glycemic control (10, 11). Dietary modification and medications (table 1) are the essential pillars for managing gastroparesis. Although prokinetic agents had been tried for a long as a treatment option for diabetic gastroparesis, results are often unsatisfactory. As these drugs are frequently associated with significant adverse reactions, their use in diabetic patients with GI symptoms should be ﬁrmly evidence-based (12-14). Thus, in this study, we compared the efficacy of two prokinetic agents in patients with diabetic gastroparesis viz. Cinitapride and levosulpiride.

## Methods

**Inclusion Criteria: **Type 2 diabetic patients aged 18-65 were diagnosed with delayed gastric emptying by gastric scintigraphy.

**Exclusion Criteria: **Type2 diabetic patients in whom gastroparesis was due to drugs like Opioid pain medicines (codeine, hydrocodone, morphine, oxycodone, tramadol, and tapentadol), antidepressants (amitriptyline, nortriptyline, venlafaxine), anticholinergics, patients on amylin analogs (e.g., pramlintide) and glucagon-like peptide 1 (e.g., exenatide), post-vagotomy / gastric surgery, smoking: (>30 pack-year) and alcoholism, thyroid disorder, parkinsonism, pregnancy, post-viral status.

**Sample Size: **It was a time-bound study for one year. Diabetes mellitus patients diagnosed with gastroparesis based on scintigraphy in 2017-2018 at tertiary care teaching hospital were 37 patients. Based on this estimate, we considered at least 40 patients to be recruited for the study period. 

**Ethical consideration: **Ethical approval was taken from institutional ethics committee (AIIMS, Rishikesh, India).

Registration number is 238/IEC/PGM/2019.

**Study Design: **A prospective, open-label randomized clinical trial was conducted in the Department of Internal Medicine at a tertiary care teaching hospital over twelve months. American Diabetes Association (ADA) criteria were used to define diabetes mellitus, patients who were more than 18 years of age having at least two gastrointestinal symptoms like nausea, vomiting, bloating/distension, or early satiety was evaluated for their possible inclusion in the study. These patients were subjected to gastric scintigraphy using Technetium 99 metastable state (Tc 99) sulphur colloid labelled standardized meal idly, and those who had delayed gastric emptying were finally included in the study. Delayed gastric emptying was indicated by retention of > 10% radiotracer activity at 4 hours after a meal. 

**Figure 1:**
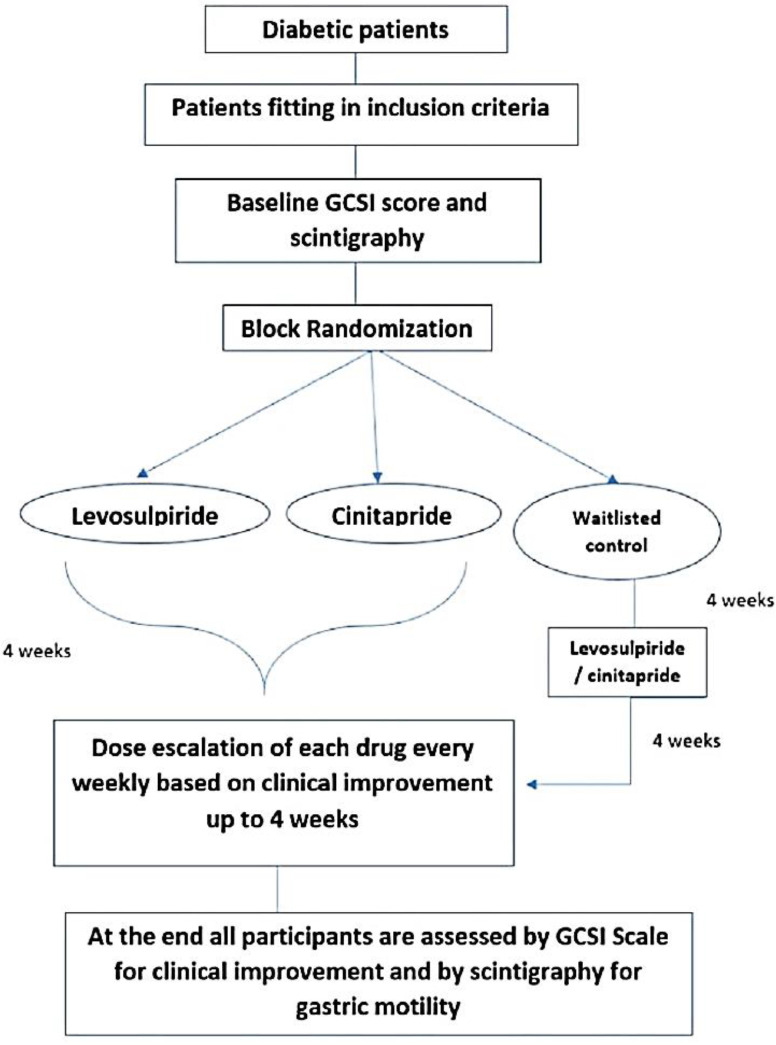
Study Design

Patients who met the inclusion criteria were included in the study. All baseline GSCI was recorded, and then they were allotted to three groups – Group-1 (Levosulpiride 25mg once daily), group-2 (Cinitapride 1mg thrice daily), and Group-3 (Waitlisted control) by block randomization based on the duration of diabetes mellitus (less than and more than five years) and follow-up done every week via telephone for four weeks. In group-1, 13 participants were on oral hypoglycemic medications and 4 participants were on insulin therapy. In group-2, 16 participants were on oral hypoglycemic drugs and 2 participants were on insulin therapy. In group-3, all 5 were on oral hypoglycemic drugs. Participants in groups-1 and 2 were given Levosulpiride and Cinitapride, respectively; along with diet and diabetic control. After four weeks duration, group-3 on diet and diabetic control only, if not improved clinically, were randomized into Levosulpiride 25mg once daily (Group 1), Cinitapride 1mg thrice daily (Group 2) for the next four weeks. At least 90% compliance will be ensured by phone once a week along with pill count. All participants in group 3 on diet and diabetic management were provided with a diet chart considering individual cultural and food habits by consulting with a dietician. They were also provided with information on foods containing high fat and fiber (reference was taken from Cleveland clinic’s diet for gastroparesis guidelines) and advised to avoid them as much as possible. Blinding could not be done as we find it challenging to obtain the two drugs of the same size, color, shape, and packet, so we considered an open-labelled study. The dose of these drugs was increased weekly up to clinical improvement, or the maximum dose was achieved. At the end of 4 weeks follow-up period, patients were reassessed by Gastroparesis Symptom Cardinal Index (GSCI) and Scintigraphy (figure 1).

**Gastric Scintigraphy**

**Patient Preparation: **Patients were advised nil by mouth for at least 6 hours or overnight, whichever was feasible. Patients were asked to avoid smoking till the study was completed. However, they were allowed to take medications with some water. 

**Figure 2:**
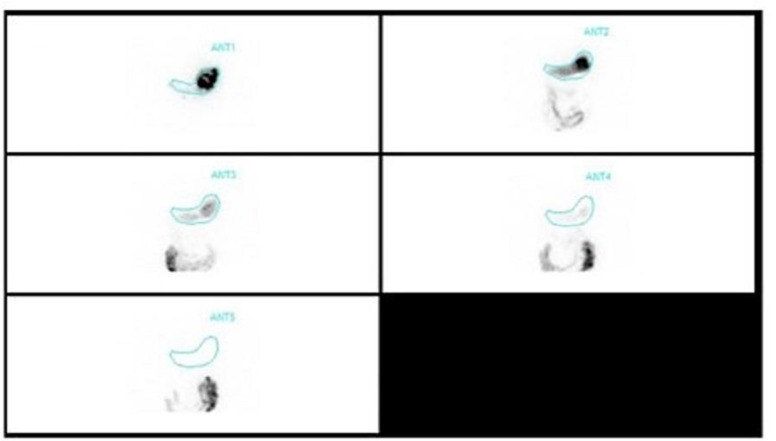
Gastric Scintigraphy – Normal Emptying: Gastric emptying scintigraphy of a 55-year male patient after the administration of radiolabelled solid Idly. Images were acquired immediately, at 30min, 1hr, 2 hrs, and 4hrs. Entire radionuclide material is seen in the first image and is seen passing gradually, at 4 hrs no tracer was seen revealing normal gastric emptying (retention ~1%). Source: With permission

**Figure 3:**
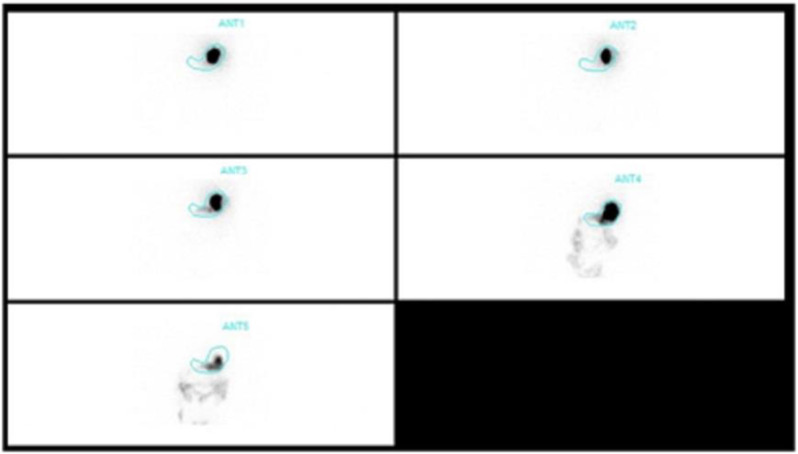
Gastric Scintigraphy – Delayed Gastric Emptying: Gastric scintigraphy of a 63-year old female patient after the administration of radiolabeled solid Idly meal. Images were acquired immediately, at 30min, 1h, 2 h, and 4h. The entire radionuclide material seen in the first image and is seen passing gradually, 4h tracer was seen revealing delayed gastric emptying (retention ~14%). Source: With permission

**Procedure: **After obtaining informed consent, patients were asked to consume radiolabelled Idly, and images were obtained immediately, 1hr, 2hr, and 4hrs after meal intake. Retention of >10% after 4hrs was taken as a cut-off to diagnose delayed gastric emptying (figures 2, 3**)**.

## Results

A total of 40 patients were included in the study (table 1). The groups were matched for age (χ2 = 1.213, p = 0.545) and gender (χ2 = 0.755, P = 0.748). Similarly, there was no significant difference between groups in terms of BMI (Kg/m2) (χ2 = 2.396, P = 0.302), FBS (mg/dl) (χ2 = 2.453, P= 0.293) and mean PPBS (mg/dl) (χ2 = 4.098, P = 0.129). Baseline parameters were comparable in all three groups (table 2) except for post prandial blood sugar (PPBS) which showed a statistically significant difference between these groups. Postprandial blood glucose of groups 1, 2 and 3 were 288 ± 47.81 mg/dl, 292.28 ± 98.3 mg/dl and 3 was 227.6 ± 64.49 mg/dl respectively. Mean HbA1c of group-1 was 7.58 ± 1.24 %, of group-2 was 7.24 ± 1.46%, of group-3 was 6.6.4 ± 0.84%. So far as complications are concerned, 9 (52.9%) had neuropathy in group-1, 9 (50 %) in group-2 and 3 (60%) in group-3. Retinopathy was seen in 7 (41.2%) in group-1, 5 (27.8%) among group-2, 2 (40%) in group-3. 

**Figure 4:**
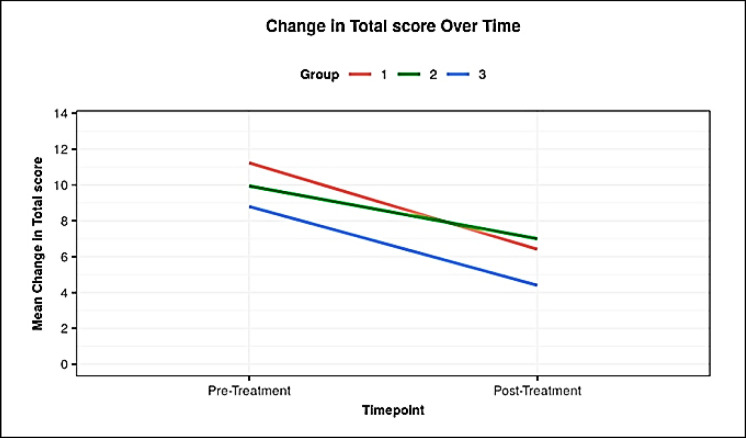
Line Diagram Depicting the Change in Total Score Over Time in the Three Groups

**Analysis of GCSI Score: **The mean (±SD) of GCSI score (pre-treatment) in groups 1, 2, and 3 was 11.24 (4.24), 9.94 (3.28), and 8.80 (2.28) years, respectively. Total pre-treatment score in these ranged from 5-19. There was no significant difference between groups in terms of total pre-treatment score (χ2 = 1.519, P = 0.468) (table 3, figure 4).

**Table 1:** Pharmacologic therapy for the treatment of gastroparesis

	**Drug Class**	**Pharmacological name**
**1.**	**Dopamine receptor antagonist**	Dopamine receptor antagonist-Metoclopramide, Domperidone, Itopride, levosulpiride.
**2.**	**5-HT4 receptor agonist**	5-HT4 receptor agonist – Cisapride, Tegaserod, prucalopride, etc
**3.**	**Motilin receptor Agonists**	Motilin receptor Agonists: Erythromycin, azithromycin
**4.**	**Cholinesterase inhibitors:**	Cholinesterase inhibitors: Neostigmine, Pyridostigmine, acotiamide
**5.**	**Ghrelin Agonists**	Ghrelin Agonists: -Remorelin ^[11]^
**6.**	**GABA receptor agonists**	GABA receptor agonists: Baclofen

GABA:Gamma-aminobutyric acid

**Table 2:** Baseline characteristics of the participants

**Parameter**	**1 (n=17)**	**2 (n=18)**	**3 (n=5)**	**P- value**
**Gender**				0.748
**Male**	9 (52.9%)	11 (61.1%)	2 (40%)	
**Female**	8 (47.1%)	7 (38.9%)	3 (60%)	
**Age (years)**	50 ± 9.02	52.17 ± 9.84	52.80 ±6.10	0.545
**31-40 yrs.**	2 (11.8%)	1 (5.6%)	0	
**41-50 yrs.**	8 (47.1%)	8 (44.4%)	2 (40%)	
**51-60 yrs.**	4 (23.5%)	6 (33.3%)	3 (60%)	
**61-70 yrs.**	3 (17.6%)	2 (11.1%)	0	
**71-80 yrs.**	0	1 (5.6%)	0	
**Height (cm)**	157.94 ± 4.78	158.22 ± 5.57	159.80 ± 4.55	0.661
**Weight (kg)**	58.59 ±6.02	61.78 ± 6.63	60.40 ± 3.65	0.314
**BMI (kg/m** ^2^ **)**	23.49 ± 2.30	24.64 ± 1.93	23.67± 1.37	0.302
**BMI**				0.514
**18.5-22.9**	7 (41.2%)	3 (16.7%)	2 (40%)	
**23- 24.9**	6 (35.5%)	7 (38.9%)	2 (40%)	
**25- 29.9**	4 (23.5%)	8 (44.4%)	1 (20%)	
**Haemoglobin (g/dL)**	10.73 ± 1.38	11.32 ± 1.55	11.79 ± 1.09	0.239
**TLC (/cu.mm)**	7.61 ± 2.34	6.39 ± 1.95	6.19 ± 0.73	0.094
**S. Urea (mg/dl)**	35.06 ± 24	37.83 ± 23.25	28.32 ± 20.08	0.220
**S. Urea**				0.203
**= 25 mg/dl**	6 (35.3%)	9 (50%)	4 (80%)	
**>25 mg/dl**	11 (64.7%)	9 (50%)	1 (20%)	
**S. Creatinine (mg/dl)**	1.02 ± 0.68	1 ± 0.40	0.92 ± 0.47	0.687
**S. Creatinine**				0.611
**= 1 mg/dl**	11 (64.7%)	9 (50%)	2 (40%)	
**>1 mg/dl**	6 (35.3%)	9 (50%)	3 (60%)	
**FBS (mg/dl)**	228.24 ± 41.66	237.89 ± 75.50	178.80 ± 62.77	0.293
**FBS**				0.237
**=126 (mg/dl)**	0	1 (5.6%)	1 (20%)	
**>126(mg/dl)**	17 (100%)	17 (94.4%)	4 (80%)	
**PPBS**	288 ± 47.81	292.28 ± 98.37	227.60 ± 64.49	0.129
**PPBS**				0.036
**=200 mg/dl**	0	1 (5.6%)	2 (40%)	
**>200 mg/dl**	17 (100%)	17 (94.4%)	3 (60%)	
**HbA1c (%)**	7.58 ± 1.24	7.24 ± 1.46	6.64 ± 0.84	0.239
**HbA1c (%)**				0.204
**<6**	1 (5.9%)	2 (11.1%)	0	
**6-7**	4 (23.5%)	5 (27.8%)	4 (80%)	
**>7**	12 (70.6%)	11 (61.1%)	1 (20%)	
**TSH (μIU/ml)**	2.82 ± 0.90	3.06 ± 0.95	2.92 ± 1.20	0.765
**FT4 (ng/ml)**	1.19 ± 0.22	1.06 ± 0.27	1.12 ± 0.18	0.289
**Duration of illness (years)**	10.71 ± 4.58	7.39 ± 3.82	6.80 ± 2.77	0.051

BMI: body mass index, PPBS: post prandial blood sugar, TSH: Thyroid stimulating hormone, FBS: Fasting Blood Sugar

**Table 3:** Comparison of the 3 subgroups in terms of total score (Pre and Post-Treatments) (n = 40)

**Total score**	**Group**			**P-value for comparison of the three groups at each of the timepoints (Kruskal Wallis Test)**
	**1**	**2**	**3**	
	**Mean (SD)**	**Mean (SD)**	**Mean (SD)**	
**Pre-Treatment**	11.24 (±4.24)	9.94 (±3.28)	8.80 (±2.28)	0.468
**Post-Treatment**	6.41 (±2.81)	7.00 (±2.45)	4.40 (±1.67)	0.126
**Overall P -value for comparison of change in Total score over time between the three groups (Generalized Estimating Equations)**	0.006			

The mean and SD of total post-treatment score in group-1 was 6.41 (±2.81) in group-2, it was 7.00 (±2.45) group-3 had a score of 4.40 (±1.67) (figure 4). There was a weak negative correlation between pre-treatment total score, BMI (kg/m^2^), FBS (mg/dl) and hba1c (%), this correlation was however not statistically significant (rho = -0.07, P= 0.675), (rho = -0.03, P = 0.874), (rho = -0.1, P= 0.529) respectively. The table 4 is depicting individual GI symptom change over time, where Levosulpiride was a step ahead of Cinitapride in symptoms such as nausea (P= 0.008), vomiting (P= 0.02), stomach fullness (P= 0.005), early satiety (P= 0.003), bloating (P=0.008) than Cinitapride.

**Analysis of Scintigraphy: **The mean (SD) of Scintigraphy (%) (pre-treatment) in group-1 was 80.59 (8.23), in group-2 was 79.22 (6.43), in group-3 was 81.80 (6.72). The mean (SD) of Scintigraphy (%) (post-treatment) in group-1 was 85.94 (5.88), in group-2 was 86.17 (3.40), in group-3 was 86.80 (4.92). There was no significant difference in the trend of scintigraphy over time among the three groups (P= 0.571) (table 5).

**Table 4:** Change in Mean of Individual Symptoms Overtime

**Gastrointestinal symptom score**	**Study group**	**Baseline score** **Mean (±SD)**	**Post-treatment** **Mean (±SD)**	**P-value (Wilcoxon test)**
**Nausea**	Group 1	1.24 (1.30)	0.71 (0.85)	0.008
	Group 2	0.50 (1.29)	0.28 (0.75)	0.371
**Retching**	Group 1	0.41 (0.87)	0.24 (0.56)	0.149
	Group 2	0.33 (0.69)	0.17 (0.38)	0.371
**Vomiting**	Group 1	0.94 (1.39)	0.35 (0.61)	0.020
	Group 2	0.17 (0.51)	0.17 (0.51)	-
**Stomach fullness**	Group 1	2.06 (1.60)	1.06 (1.20)	0.005
	Group 2	1.83 (1.54)	1.44 (1.34)	0.048
**Early satiety**	Group 1	2.65 (1.54)	1.41 (1.06)	0.003
	Group 2	2.11 (1.53)	1.44 (1.34)	0.012
**Fullness after eating**	Group 1	1.06 (1.25)	0.82 (0.88)	0.346
	Group 2	1.56 (1.58)	0.78 (0.94)	0.010
**Loss of appetite**	Group 1	0.65 (1.00)	0.47 (0.72)	0.188
	Group 2	0.78 (1.06)	0.61 (0.92)	0.083
**Bloating**	Group 1	1.76 (1.48)	0.88 (0.93)	0.008
	Group 2	2.44 (1.29)	1.94 (1.30)	0.018
**Belly visible large**	Group 1	0.59 (0.80)	0.59 (0.80)	-
	Group 2	0.22 (0.55)	0.17 (0.51)	0.331

Group 1=L.

Change in mean overall and individual gastrointestinal symptom score before and after treatment between Levosulpiride and Cinitapride

**Table 5:** Comparison of three Groups in Terms of Change in Scintigraphy Over Time

**Scintigraphy (%)**	**Group**			**P-value for comparison of the three groups at each of the timepoints (Kruskal Wallis Test)**
	**1**	**2**	**3**	
	**Mean (SD)**	**Mean (SD)**	**Mean (SD)**	
**Pre-Treatment**	80.59 (8.23)	79.22 (6.43)	81.80 (6.72)	0.561
**Post-Treatment**	85.94 (5.88)	86.17 (3.40)	86.80 (4.92)	0.838
**Overall P -value for comparison of change in Scintigraphy (%) over time between the three groups (Generalized Estimating Equations)**	0.571			

## Discussion

Present study found Levosulpiride more effective than Cinitapride in improving individual symptoms like nausea, vomiting, stomach fullness, and early satiety. Both postprandial and pre-prandial blood glucose levels depend on gut absorption, meal content, gastric emptying, insulin secretion, etc.; long-standing uncontrolled diabetes can lead to gastroparesis (15, 16). Apart from being a hurdle for optimal glycemic control, diabetic gastroparesis also leads to complications in advanced stages such as malnutrition, electrolyte imbalance, bezoar formation (17, 18). 

Among the patients with gastroparesis, survival was significantly lower, especially in patients with diabetic gastroparesis rather than idiopathic gastroparesis (19). Metoclopramide is one of the oldest and most commonly used agents in the management of diabetic gastroparesis (20). This is the first study to the best of our knowledge comparing the efficacy of Levosulpiride and Cinitapride in diabetic gastroparesis. In our study of 40 patients, 22 (55%) were males, and 18 (45%) were females. Determination of epidemiology of diabetic gastroparesis is a difficult task because of fewer community-based studies due to lack of scintigraphy facilities, which is essential for making a definite diagnosis of gastroparesis. The only landmark study conducted in Olmsted County, Minnesota, under the Rochester Epidemiology Project's aegis, is the most extensive study on the epidemiology of diabetic gastroparesis. They collected data from patients who sorted medical care rather than at the community level and concluded that the age-adjusted prevalence of definite gastroparesis was approximately fourfold higher in women than in men (21). 

There was no statistically significant association between duration of diabetes, levels of hba1c, and diabetic gastroparesis symptom severity in the present study. However, an article published by Muhammad Umer Nisar et al. showed a 16.9 times increased risk of developing neuropathy in patients with hba1c > 6.5% (22). More prolonged diabetes mellitus is associated with an increased level of advanced glycosylation end products, endothelial injury, and oxidative species release (23, 24). A meta-analysis conducted by Yeon-Ji Kim et al. from the Republic of Korea showed in his analysis that there was a statistically significant betterment in hba1c levels with prokinetic agents among four out of 5 studies (25). 

In our study, 5/13 patients showed symptomatic improvement who were enrolled in the diet and lifestyle group. The remaining seven patients were randomly assigned to the Levosulpiride and Cinitapride groups after the nonpharmacological improvement was not there. Patients were provided with a diet chart that contained multiple small low-fat, low fiber meals per day (5-6/day). As high fat and poorly digestible fiber need good and effective antral motility, and high fiber can increase the risk of bezoar formation (26). Carol Rees Parrish et al., in their article, laid many recommendations for proper diet management in patients with diabetic gastroparesis (27). 

These guidelines were laid to increase gastroparesis patients' nutritional status as often these patients are at increased risk of malnutrition and weight loss, further making the management of diabetes and maintaining general health difficult in these patients. There was no statistically significant difference in total gastroparesis symptoms scores and scintigraphy in the three groups. However, Levosulpiride is a step ahead in clinical improvement of nausea, vomiting, early satiety, stomach fullness, and bloating. This improvement could be attributed to Levosulpiride's action on D2 receptors in the enteric nervous system and the chemoreceptor trigger zone (28). 

Previous studies comparing Levosulpiride and Cisapride conducted by C. Mansi et al. showed that both Cisapride and Levosulpiride were comparable gastric emptying, and there was no statistically significant improvement in total symptom score (29). 

However, Levosulpiride performed well in symptoms such as nausea, vomiting, early satiety compared to Cisapride. None of the patients in our study reported adverse drug reactions. Parveen Malhotra et al. observed that Levosulpiride and proton pump inhibitors provided a significant clinical improvement and glucose homeostasis (30). 

They also showed a significant reduction in gastric emptying compared to placebo. Prokinetics, apart from helping in symptomatic relief, showed good improvement in diabetic control as well. The study's limitations are the small sample size, open label, and effect of glycemic control with the use of prokinetic drugs was not studied. Follow-up was done only for four weeks; hence, long-term effects and recurrence of improved symptoms during the study were not evaluated.

The management of diabetic gastroparesis is very challenging. With advances in understanding, the pathophysiological changes, the scope for developing newer drugs is increasing. Levosulpiride is better than Cinitapride in improving the symptoms of diabetic gastroparesis apart from diet and diabetic control but no significant effect on gastric scintigraphy. Prokinetics, apart from helping in symptomatic relief, showed good improvement in diabetic control as well.
